# Allopregnanolone as an Adjunct Therapy to Midazolam is More Effective Than Midazolam Alone in Suppressing Soman‐Induced Status Epilepticus in Male Rats

**DOI:** 10.1111/cns.70215

**Published:** 2025-02-28

**Authors:** Peter M. Andrew, Jeremy A. MacMahon, Xiuzhen Liu, Naomi H. Saito, Kyle E. Berger, Julia E. Morgan, Ashish Dhir, Danielle J. Harvey, Hilary S. McCarren, Michael A. Rogawski, Pamela J. Lein

**Affiliations:** ^1^ Department of Molecular Biosciences University of California, Davis Davis California USA; ^2^ Department of Public Health Sciences, School of Medicine University of California, Davis Davis California USA; ^3^ Neuroscience Department, Medical Toxicology Research Division US Army Medical Research Institute of Chemical Defense Aberdeen Maryland USA; ^4^ Department of Neurology, School of Medicine University of California, Davis Sacramento California USA; ^5^ Department of Pharmacology, School of Medicine University of California, Davis Sacramento California USA

**Keywords:** benzodiazepine, chemical threat agents, neurosteroid, organophosphate, polytherapy, seizure

## Abstract

**Aims:**

Humans and animals acutely intoxicated with the organophosphate soman can develop sustained status epilepticus (SE) that rapidly becomes refractory to benzodiazepines. We compared the antiseizure efficacy of midazolam, a current standard of care treatment for OP‐induced SE, versus combined therapy with midazolam and allopregnanolone (ALLO) in a rat model of soman‐induced SE.

**Methods:**

Soman‐intoxicated male rats with robust seizure behavior and high‐amplitude electroencephalographic (EEG) activity were administered midazolam (0.65 mg, i.m.) 20 min after seizure initiation and 10 min later either a second dose of midazolam or ALLO (12 or 24 mg/kg, i.m.). Seizure behavior and EEG were monitored for 4 h after treatment. Brains were collected at the end of the monitoring period for histological analyses.

**Results:**

Animals receiving 2 doses of midazolam exhibited persistent SE. Sequential dosing with midazolam followed by ALLO suppressed electrographic seizure activity. The combination therapy also significantly reduced soman‐induced neurodegeneration and neuroinflammation compared to 2 doses of midazolam. High but not low dose ALLO was associated with transitory and reversible respiratory compromise during the 1 h period after dosing.

**Conclusions:**

Treatment with midazolam followed by ALLO was more effective than 2 doses of midazolam in suppressing benzodiazepine‐refractory, soman‐induced SE, and in mitigating its acute neuropathological consequences.

## Graphical abstract

Acute poisoning with the organophosphate, soman, triggers status epilepticus that rapidly becomes refractory to standard of care treatment with midazolam (MDZ). Combined treatment with MDZ and allopregnanolone (ALLO) significantly suppresses seizure activity, which is associated with attenuated neurodegeneration and neuroinflammation.

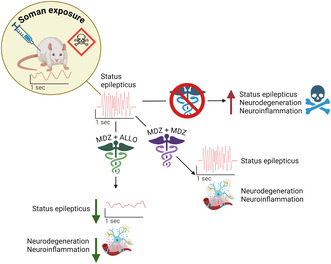

## 1 Introduction

Organophosphate (OPs) pesticides and nerve agents are among the most potent chemical threats. Acute intoxication with OPs can produce a life‐threatening toxidrome known as cholinergic crisis, characterized by parasympathomimetic symptoms, seizures that can progress to status epilepticus (SE), and respiratory paralysis [1]. Human survivors of acute OP intoxication often exhibit structural changes in white matter [2], EEG abnormalities, and deficits in psychomotor function and memory [3, 4]. OP intoxication in animals causes irreversible neuronal damage, a rapid and robust neuroinflammatory response that can persist for months, behavioral deficits, and the development of spontaneous recurrent seizures (SRS) [5, 6, 7, 8].

Current medical countermeasures for OP poisoning include the muscarinic agonist atropine and an oxime cholinesterase reactivator to address peripheral symptoms and a benzodiazepine to treat acute seizures [1]. The benzodiazepine midazolam (MDZ) has been approved by the FDA as an autoinjectable treatment for SE and is a first‐line treatment for OP‐induced seizures [1, 9]. If administered within 10 min after initiation of SE, benzodiazepines can effectively terminate the SE. However, in many scenarios of acute human poisoning with OPs, it is expected that > 30 min to hours will elapse before medical interventions are administered [10, 11]. Benzodiazepines are generally ineffective in terminating established SE 30 min or more after seizure onset. Given the extensive research demonstrating a positive correlation between the severity and duration of OP‐induced seizures and subsequent neuropathology [12, 13, 14], rapid termination of OP‐induced benzodiazepine‐refractory SE is a key therapeutic goal for protecting against the chronic adverse neurological consequences of acute OP intoxication [9, 15, 16].

Refractoriness to the antiseizure efficacy of benzodiazepines is thought to be due to the reduced trafficking and internalization of synaptic GABA_A_ receptors that occurs during prolonged seizure activity [17, 18]. In particular, there is reduced surface expression of γ2 GABA_A_ receptor subunits, which are required for GABA_A_ receptor clustering at synapses and for benzodiazepine action. In addition, SE is associated with impaired neuronal chloride extrusion due to reduced expression of the potassium–chloride co‐transporter KCC2, which leads to a depolarizing shift in the chloride electrochemical gradient so that GABA_A_ receptor activation becomes excitatory [19]. Benzodiazepines, including MDZ, act exclusively on synaptic GABA_A_ receptors. Neuroactive steroids, which enhance activation not only of synaptic GABA_A_ receptors but also extrasynaptic receptors that are not internalized with prolonged seizure activity, have emerged as promising therapeutic candidates for the management of benzodiazepine‐refractory SE [20, 21, 22]. One such neuroactive steroid is allopregnanolone (ALLO), an endogenous metabolite of progesterone currently approved for treating post‐partum depression in women [23]. ALLO has been shown to terminate SE in humans and preclinical models [20, 24, 25], including SE induced by the OPs diisopropylfluorophosphate (DFP) and sarin [26, 27, 28, 29, 30]. The goal of the present study is to assess the efficacy of adjunctive ALLO in attenuating the acute electrophysiological and neuropathological changes caused by the OP nerve agent soman. Acute soman intoxication causes robust, benzodiazepine‐resistant SE associated with pronounced neuropathology and neurological consequences [31]. The current recommendation for the treatment of nerve agent seizures is to administer a benzodiazepine, either diazepam or MDZ, followed by a second dose if the first dose fails to terminate seizures (see, https://chemm.hhs.gov). Here, we assessed the efficacy of substituting ALLO for the second benzodiazepine dose in the treatment of refractory soman‐induced seizures. Our findings demonstrate that sequential treatment with MDZ and ALLO is superior to treatment with 2 doses of MDZ in suppressing soman‐induced SE and indicate that ALLO can partially mitigate the acute neuropathological consequences of soman‐induced SE.

## 2 Materials and Methods

### 2.1 Chemicals

All drugs were diluted with sterile saline unless otherwise noted. Soman (synthesized by the U.S. Army Combat Capabilities Development Command Chemical Biological Center, Aberdeen Proving Ground, MD) was diluted to 360 μg/mL; the oxime HI‐6 (Kalexsyn, Kalamazoo, MI; 97.8% pure), 250 mg/mL; atropine methyl nitrate (AMN; Sigma‐Aldrich; St. Louis, MO; 98% pure), 4 mg/mL; and MDZ (Hospira, Lake Forest, IL; 97% pure), 2.0 mg/mL. Atropine sulfate (Medisca, Irving, TX; 97% pure) was diluted to 1.8 mg/mL and then admixed with 2‐PAM (ScienceLab.com, Houston, TX; 99.3% pure) diluted to 100 mg/mL. ALLO (99.9% pure), synthesized under contract by SAFC Pharma (Madison, WI), was administered as an aqueous 12 mg/mL solution with 40% w/v hydroxypropyl‐β‐cyclodextrin (Captisol, CyDex) and 0.9% w/v NaCl.

### 2.2 Animals

All experiments involving animals were approved by the Institutional Animal Care and Use Committee (IACUC) at the United States Army Medical Research Institute of Chemical Defense. All procedures were conducted in accordance with the Guide for the Care and Use of Laboratory Animals and the Animal Welfare Act of 1966. Adult male Sprague Dawley rats (*n* = 48) purchased from Charles River Laboratories (Wilmington, MA) were single housed in a temperature (20°C–26°C) and humidity (30%–70%) controlled environment on a 12:12 h light dark cycle and were provided food and water *ad libitum*.

**FIGURE 1:**
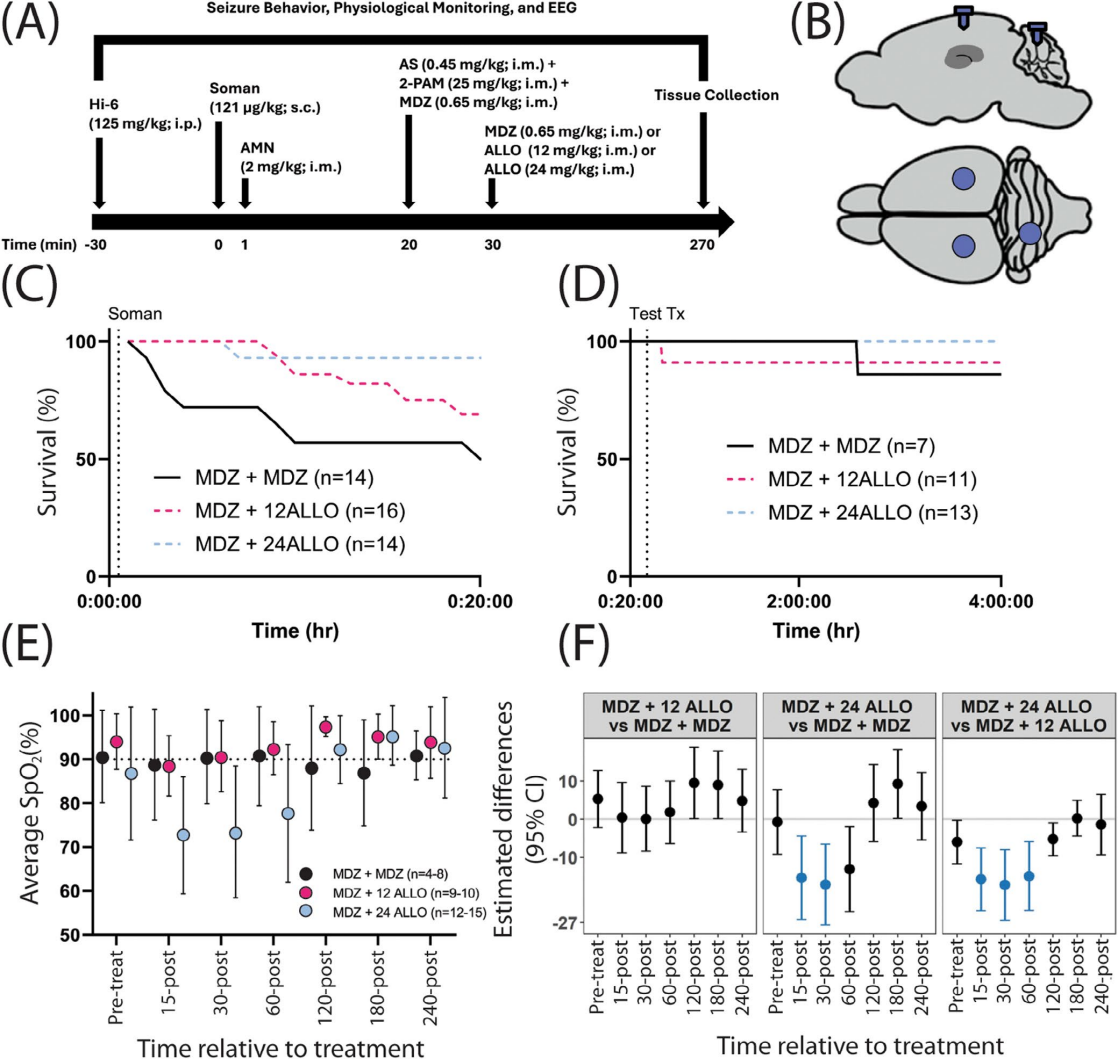
(A) Adult male Sprague Dawley rats were implanted with EEG headpieces and allowed to recover for 5–7 d. On the day of experimentation, all animals were given the oxime HI‐6 (125 mg/kg, i.p.) 30 min before exposure to soman (121 μg/kg, s.c.) and treated with atropine methyl nitrate (AMN; 2 mg/kg, i.m.) 1 min after exposure. At 20 min after seizure onset, all animals received atropine sulfate (AS; 0.45 mg/kg, i.m), pralidoxime (2‐PAM; 25 mg/kg, i.m.), and midazolam (MDZ; 0.65 mg/kg, i.m.), and 10 min later, either a second injection of MDZ (0.65 mg/kg, i.m.) or allopregnanolone (ALLO) at either 12 or 24 mg/kg, i.m. EEG was recorded starting 30 min prior to continuing until 240 min after antiseizure drug administration. At the end of the recording period, animals were euthanized to harvest brain tissue for histological analyses of neurodegeneration and inflammation. (B) Placement of screws for EEG monitoring; one screw over each hemisphere at 4 mm posterior, +/− 4 mm mediolateral (ML); a third ground screw is placed over the cerebellum at approximately 10 mm posterior, 2 mm ML. (C) Kaplan–Meier curves showing survival during the 20 min period between soman administration and initial treatment with MDZ. (D) Survival in the treatment groups from time of initial treatment to end of observation period. (E) Mean ± SD arterial blood oxygen saturation (SpO_2_) values as measured at various times during the observation period in the three treatment groups. SpO_2_ levels below 90% (indicated by the horizontal dotted line) indicate hypoxemia. (F) Statistical analyses of SpO_2_ data. Data are presented as mean differences (dot) with 95% confidence interval (bar) of average SpO_2_ (%) between two groups (identified at the top of each box) (*n* = 4–15 animals per group). The horizontal line in each box corresponds to a difference of 0, which would indicate no difference between the two groups being compared. Confidence intervals that do not include 0 indicate a statistically significant difference between the two groups being compared; CI bars colored blue indicate significant differences that survived false discovery rate (FDR) correction.

Prior to experimentation, rats were randomly assigned to one of three antiseizure treatments: (1) two sequential doses of 0.65 mg/kg MDZ (MDZ + MDZ); (2) 0.65 mg/kg MDZ and 12 mg/kg ALLO (MDZ + 12 ALLO); or (3) 0.65 mg/kg MDZ and 24 mg/kg ALLO (MDZ + 24 ALLO) (Figure 1A). Cohorts of 8 rats at a time were exposed to nerve agent and treatment. Body weights on the day of exposure ranged from 323 to 416 g. Sixteen rats intoxicated with soman either died prior to antiseizure treatment or were excluded due to technical difficulties. Final sample sizes were MDZ + MDZ (*n* = 8), MDZ + 12 ALLO (*n* = 11), and MDZ + 24 ALLO (*n* = 13).

### 2.3 Nerve Agent Exposure

On the day of exposure, rats were placed in 25 × 25 × 45 cm plastic recording chambers and connected to the EEG recording system via a cable on a commutator that allowed for 360^o^ movement. Approximately 30 min of baseline activity was recorded. Next, rats were pretreated with 125 mg/kg, i.p. HI‐6, followed 30 min later by 121 μg/kg, s.c. soman. One minute after soman, rats received 2 mg/kg, i.m. AMN to mitigate the toxic peripheral effects of nerve agent so that sufficient numbers of rats would survive. This protocol elicited SE in 100% of the rats. Onset of SE was visually scored by a trained observer during the experiment and confirmed post hoc by a second trained observer. Twenty min after the onset of SE, rats received 0.45 mg/kg, i.m. atropine sulfate, 25 mg/kg, i.m. 2‐PAM, and 0.65 mg/kg, i.m. MDZ. Ten min later, they were given either a second dose of 0.65 mg/kg i.m. midazolam or ALLO at 12 mg/kg or 24 mg/kg, i.m. EEG recording continued for an additional 4 h (Figure 1A). This dosing regimen is consistent with the standard of care for treating OP‐induced seizures (U.S. Department of Health and Human Services Chemical Hazards Emergency Medical Management; https://chemm.nlm.nih.gov/), which specifies that OP‐induced SE be treated prehospital with a single autoinjector of MDZ (10 mg, i.m.), and if seizures do not cease, a second autoinjector of MDZ be administered 10 min later. The dose of MDZ was selected to approximate the plasma exposure obtained in humans following a 10 mg dose of Seizalam (A. Dhir, C.‐Y. Wu and M.A Rogawski, in preparation).

### 2.4 Seizure and Physiological Monitoring

Rats were implanted with cortical EEG electrodes and headpiece plugs 5–7 d before nerve agent exposure (Figure 1B). Approximately 15 min prior to surgery and again ~24 h after surgery, they received 1 mg/kg s.c. meloxicam (Pivetal, Liberty, MO) for analgesia. Surgery was performed as previously described [32]. Anesthesia was induced with 3%–5% isoflurane (Baxter Healthcare, Deerfield, IL) in oxygen at a flow rate of 0.5–1 L/min and maintained at 1%–3%. Pulse oximetry was monitored, and body temperature was regulated throughout the procedure using a PhysioSuite system (Kent Scientific, Torrington, CT).

EEG data were collected using an MP160 data acquisition system with AcqKnowledge software (BIOPAC Systems Inc.; Goleta, CA) at a sampling rate of 500 Hz. Additional physiological measurements and observations were performed at the following time points with respect to treatment with either the second dose of MDZ or the dose of ALLO: immediately pre‐treatment and 15 and 30 min, 1, 2, 3, and 4 h post‐treatment. Oxygen saturation was measured using a MouseOx system (Starr Life Sciences Corp.,; Oakmont, PA).

Analysis of EEG activity was performed using NeuroScore analytical software (version 3.3.9, DSI). Data were pre‐processed through a 1 Hz high‐pass filter to remove movement artifact. Spike amplitude threshold was defined as an amplitude greater than four‐times the standard deviation of the average baseline oscillatory amplitude. Each animal's spike threshold was applied across the entire recording period to identify individual spikes. Spike count data were binned into 1‐min epochs. A baseline spike rate was determined for each animal by averaging spike count data over the recording period prior to soman administration. Spike rate for the entire recording was normalized to baseline rate for data analysis and presentation.

To analyze EEG power, data were binned into 1‐min epochs and power spectra were obtained using the Periodogram Power Bands function in NeuroScore for frequencies from 0.5 to 125 Hz. Power bands were summed together for an absolute power calculation. Baseline power was calculated as the average absolute power of 1‐min epochs prior to soman administration and was used to normalize power across the entire recording period and account for inter‐animal variability. For ease of data visualization, metrics of EEG activity were normalized first to baseline values and then plotted as the ratio of baseline‐normalized values to the maximal value obtained over the recording period.

### 2.5 Neuropathological Analysis

Approximately 4 h after anti‐seizure treatment, rats were deeply anesthetized with > 75 mg/kg i.p. sodium pentobarbital (Voltech, Dearborn, MI) and then transcardially perfused with phosphate buffered saline followed by 4% paraformaldehyde (Sigma‐Aldrich). Brains were then removed, cut into 2‐mm coronal sections using a rat brain matrix and post‐fixed in 4% paraformaldehyde for 24 h. Brain sections were transferred to 30% sucrose in PBS for 48 h before being embedded into O.C.T compound (Thermo Fisher Scientific,; Waltham, MA, USA) and snap frozen in a dry ice/methanol mixture. Tissue blocks were cryosectioned into 10‐μm thick slices using a Microm HM550 cryostat (Thermo Fisher Scientific) and mounted onto Superfrost Plus microscope glass slides (Thermo Fisher Scientific) and stored at −20°C until further processed for immunohistochemistry.

Immunostaining was performed as previously described [6]; primary and secondary antibodies are described in Table S1. Immunostained sections were mounted in ProLong^tm^ Gold Antifade Mountant with DAPI (Invitrogen,; Waltham, MA). Fluoro‐Jade C (FJC, AG325, Millipore Sigma,; Burlington, MA) staining was performed as previously described [33]. Fluorescence was quantified in four brain regions (thalamus, piriform cortex/amygdala, hippocampus, somatosensory cortex) as previously described [6]. Briefly, for sections co‐labeled for GFAP/S100β or IBA1/CD68, immunopositive cells were quantified in two consecutive sections using MetaXpress High‐Content Image Acquisition and Analysis software (version 5.3, Molecular Devices, San Jose, CA) or ImageJ (version 1.48, National Institutes of Health, Bethesda, MD). Positive staining was identified as fluorescence intensity that was twice that of the background fluorescence levels observed in negative control images. To minimize bias, image acquisition and analyses were performed by a single experimenter who was blinded to treatment group.

### 2.6 Data and Statistical Analysis

The percentage of animals in SE following treatment and percentage of animals that experienced recurrence of SE was analyzed using the Fisher Exact test. The percentage of time seizing post‐administration of test therapeutics was evaluated using the Kruskal–Wallis test with Dunn's multiple comparisons. Both analyses were completed using Prism 10.1.0 (GraphPad Software, La Jolla, CA, USA).

SpO_2_ levels (%) were analyzed using mixed effects regression, including animal‐specific random effects. Primary factors of interest were group and time point. Interactions between factors were considered, and the best model was chosen using Akaike information criterion (AIC). Contrasts for differences between treatment groups by time point were constructed and tested using a Wald test. The Benjamini–Hochberg false discovery rate (FDR) was used to account for multiple comparisons of SpO_2_ between groups at the different time points.

The primary EEG outcomes were spike rate and absolute power measured at five key time intervals: (1) pre‐exposure to soman; (2) initial response to soman; (3) response to initial dose of MDZ at 20 min after seizure initiation; (4) response to second antiseizure treatment at 30 min after seizure initiation; and (5) late treatment window. Outcomes were averaged over each interval of interest for each animal. Animals that died before receiving the second antiseizure treatment were excluded (*n* = 13). Mixed effects models, similar to those described for SpO_2_ levels were used. All outcomes were transformed using the natural logarithm to better meet the assumptions of the model.

The primary histological outcomes were region‐specific FJC, GFAP, S100β, IBA1, and CD68 labelling, and GFAP/S100β and IBA1/CD68 colocalization. Mixed effects models, similar to those described above for SpO_2_ levels were used. Primary factors of interest included treatment and brain region. All outcomes except for GFAP and S100β were transformed using the natural logarithm to better meet the assumptions of the model.

For EEG and histological data, results are presented as geometric mean ratios (GMR) between two groups with the exception of the SpO_2_, GFAP, and S100β immunohistochemical data, which are presented as the mean differences between groups. In the figures representing GMR, point estimates of the ratios and 95% confidence intervals are presented (and specific values are provided in Tables S2–S7). When the confidence interval for the GMR includes 1 or the mean difference includes 0, there is no statistical evidence of a difference between the two groups being compared.

All models used in analyses had an underlying assumption of normality of the residuals from the models. Diagnostic plots, including residual plots and QQ‐plots were used to assess the validity of these assumptions. When assumptions were violated, outcomes were transformed using the natural logarithm and model assumptions were reassessed. This transformation, when needed, fixed the issues with the underlying model assumptions. All analyses were performed using SAS software, version 9.4 and alpha was set at 0.05. Comparisons remained statistically significant after the FDR correction, unless otherwise stated.

## 3 Results

### 3.1 Soman‐Induced SE and Mortality

Mean latency to seizure onset following soman exposure was 5.58 ± 3.15 min (mean ± SD). All soman‐intoxicated animals entered SE as defined by the American Clinical Neurophysiology Society: seizures with a frequency of ≥ 2.5 Hz for ≥ 10 s that last for ≥ 10 min or have a total duration of ≥ 20% of any given 60‐min period [34]. Across all groups, 12 out of 48 animals (25%) died prior to MDZ administration at 20 min post‐soman (Figure 1C). Another four animals died prior to or immediately after the second administration of an antiseizure drug at 30 min after seizure initiation (Figure 1C). No significant differences in mortality were observed between experimental groups following administration of antiseizure therapeutic at 30 min after initiation of seizure activity (Figure 1D). Significant, but transient, hypoxia was detected in animals treated with MDZ and 24 mg/kg ALLO (Figure 1E,F).

### 3.2 Effects of ALLO on Seizure Activity

**FIGURE 2:**
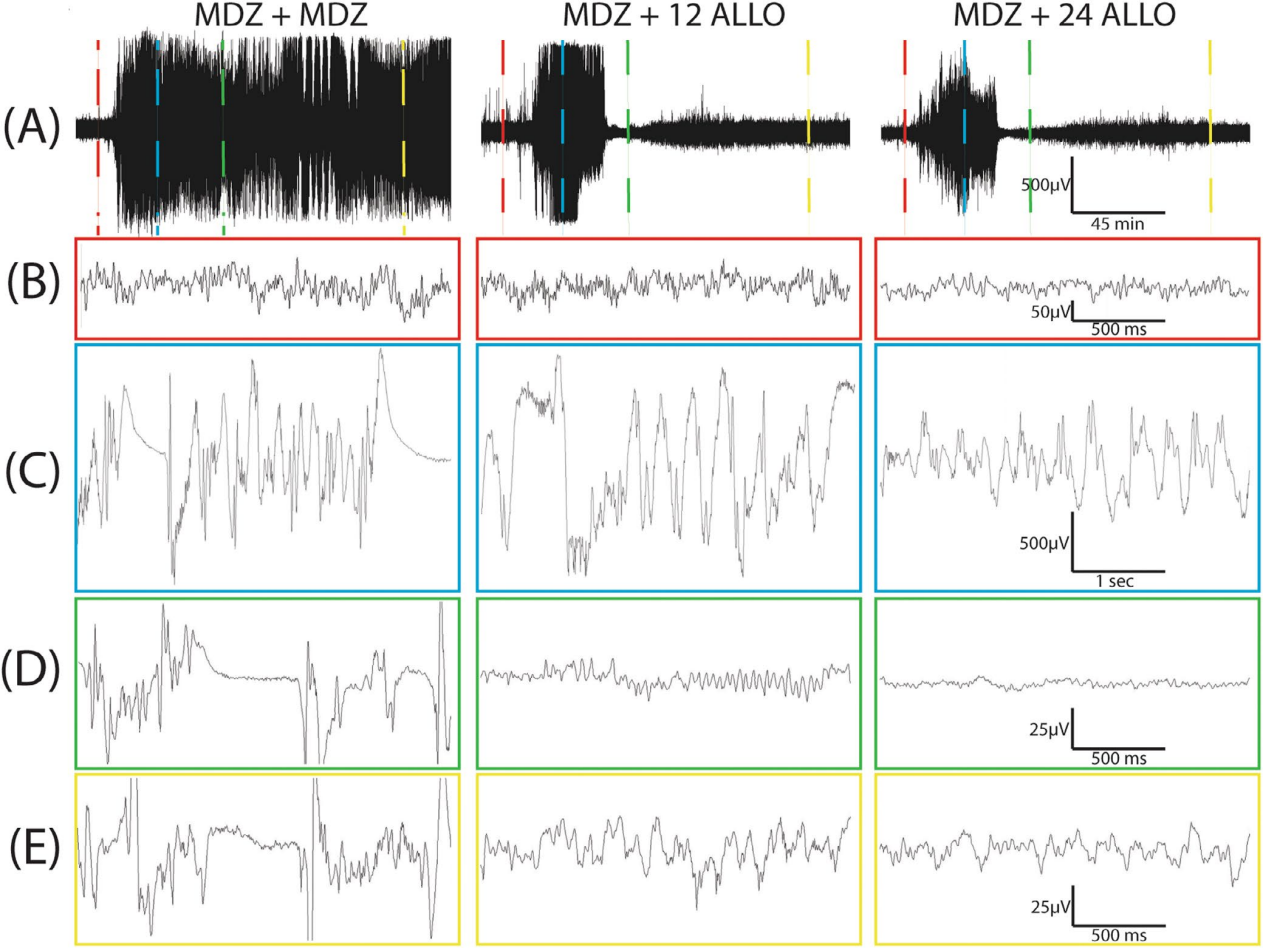
(A) Representative EEG traces from an animal in each of the groups receiving: (1) MDZ + MDZ, (2) MDZ + ALLO 12 mg/kg, (3) MDZ + ALLO 24 mg/kg). (B–E) Samples of the EEG traces on an expanded time scale. Colored vertical dashed lines in (A) indicate positions of the samples shown in the colored boxes below each trace in panels (B–E). (B) Representative traces of baseline EEG activity prior to soman exposure. (C) Representative traces following soman exposure. (D, E) Representative traces at 2 time points after administration of the antiseizure drug pairs.

**FIGURE 3:**
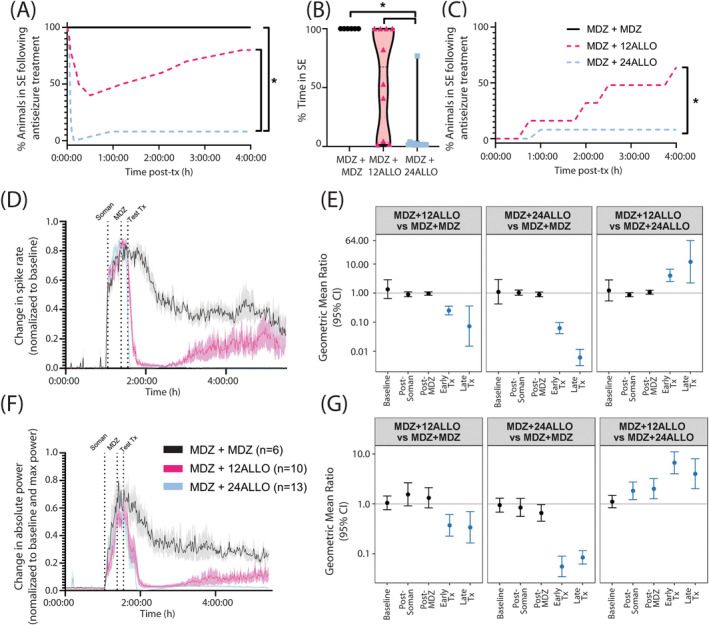
(A) The percentage of animals that remained in SE as determined by behavioral seizure scores presented as a function of time after treatment (tx) with MDZ + MDZ (black solid line), MDZ + ALLO 12 mg/kg (red dashed line), MDZ + ALLO 24 mg/kg (blue dashed line) (*n* = 6–13 per group). * Indicates a significant difference between groups as determined by Fisher Exact test (*p* < 0.05). (B) The percentage of time animals were in SE as determined using behavioral seizure scores following treatment with antiseizure drug, calculated as time to SE termination plus time in SE after recurrence as applicable. Data are presented as violin plots, with each individual point within the plot representing a single animal treated with MDZ + MDZ (black), MDZ + ALLO 12 mg/kg (magenta) or MDZ + ALLO 24 mg/kg (cyan) (*n* = 6–13 per group). * Indicates a significant difference between groups as determined by Kruskal‐Wallis non‐parametric one‐way ANOVA (*p* < 0.05). (C) The percentage of animals that experienced recurrence of SE following termination of seizure activity with ALLO 12 mg/kg (magenta; *n* = 5) or ALLO 24 mg/kg (cyan; *n* = 13). No animals in the MDZ + MDZ group experienced termination of seizure activity; therefore, the MDZ + MDZ group is not included in panel (C). * Indicates a significant difference between groups as determined by Fisher Exact test (*p* < 0.05). Antiseizure treatment effects on (D and E) change in spike rate and (F and G) absolute EEG power over the recording period in animals treated with MDZ + MDZ (black lines), MDZ + ALLO 12 mg/kg (magenta lines), or MDZ + ALLO 24 mg/kg (cyan lines). (D and F) For each animal, data were normalized to baseline and are presented as the mean ± SD (shaded regions) (*n* = 6–13 per group). The times at which soman and antiseizure treatments (Tx) were administered are indicated by vertical dotted lines. (E and G) Geometric mean ratio (GMR, dot) and 95% confidence interval (CI, bars) of (E) spike rates and (G) absolute power between two treatment groups (identified at the top of each box) (*n* = 6–13 per group). The horizontal line in each box corresponds to a GMR of 1.0, which would indicate the geometric mean of the parameter was identical in the two groups being compared. Confidence intervals that do not include 1.0 indicate a statistically significant difference between the two groups being compared; CI bars colored blue indicate the significant difference survived false discovery rate (FDR) correction.

Treatment with two sequential doses of MDZ did not alter EEG outcomes; in contrast, treatment with either dose of ALLO following the initial dose of MDZ significantly attenuated electrographic activity (Figure 2). Assessment of seizure behavior using a modified Racine scale [35] indicated differences between groups in the time to and persistence of SE termination following administration of the second dose of antiseizure therapeutic (Figure 3). Animals treated with two consecutive doses of MDZ (MDZ + MDZ) did not exit SE during the 4 h observation period. Administration of ALLO at 12 mg/kg (12 ALLO) terminated behavioral seizures in 6 of 11 animals, while the remaining animals (5/11) continued in SE throughout the monitoring period. Behavioral seizures were terminated in all the animals treated with ALLO at 24 mg/kg (24 ALLO) within the first hour (Figure 3A). Differences in seizure control were also reflected in the percentage of time animals spent seizing after administration of test therapeutics. The 24 ALLO group spent less time in SE than the animals in either the MDZ + MDZ or 12 ALLO groups (*p* < 0.001 and *p* = 0.02, respectively) (Figure 3B). Persistence of SE control similarly varied with antiseizure treatment: SE recurrence was observed in 4 of 6 animals in the 12 ALLO group, while SE recurrence was observed in 1 of 13 animals in the 24 ALLO group (*p* = 0.0173) (Figure 3C).

According to the current ACNS criteria [34], electrographic SE is associated with electrographic seizures that are defined as epileptiform discharges (spikes, polyspikes, and sharp waves) at a high rate (> 2.5 Hz) as well as more complex patterns. We used a spike detection algorithm that provides a rough assessment of epileptiform discharge rate as one approximate marker of SE. There were no significant differences in spike rate between groups prior to soman administration (baseline; *p* > 0.41), in the immediate interval after soman administration (*p* > 0.12), or after the initial MDZ dose (*p* > 0.25) (Figure 3D,E). ALLO treatment significantly suppressed spike rate compared to MDZ only treatment during the early post experimental treatment window (Figure 3E). This relationship held during the late treatment window, with spike rate remaining significantly lower in the MDZ + 12 ALLO and MDZ + 24 ALLO groups compared to the MDZ + MDZ group (Figure 3E). Additionally, there is evidence of dose‐dependency in ALLO treatment: the spike rate was significantly higher in the MDZ + 12 ALLO group compared to the MDZ + 24 ALLO group during both the early and late treatment windows (Figure 3E).

EEG power is a measure of oscillatory amplitude and frequency [36], and increases in this EEG metric are commonly used as a marker of SE. There were no significant differences in absolute power between groups prior to soman administration (baseline or pre‐soman interval; *p* > 0.46), confirming no group differences in basal EEG power (Figure 3F,G). Soman increased power in all groups. Comparisons between the two ALLO treatment groups indicated that absolute power was elevated post‐soman in the MDZ + 12 ALLO compared to the MDZ + 24 ALLO group, but there were no significant differences between either ALLO group and the MDZ + MDZ group (*p* > 0.1) (Figure 3G). Additionally, absolute power was significantly greater after the initial dose of MDZ in the MDZ + 12 ALLO group compared to the MDZ + 24 ALLO group (Figure 3G), indicating that animals in this group were not as responsive to the initial dose of MDZ as animals in the MDZ + 24 ALLO group. Following ALLO intervention, power in both MDZ + ALLO groups was significantly suppressed compared to the MDZ + MDZ group in both the early treatment windows (Figure 3G). Similar to spike rate observations, absolute power was significantly elevated in the MDZ + 12 ALLO group compared to the MDZ + 24 ALLO group at the early and late treatment windows (Figure 3G).

### 3.3 Neuropathological Outcomes

**FIGURE 4:**
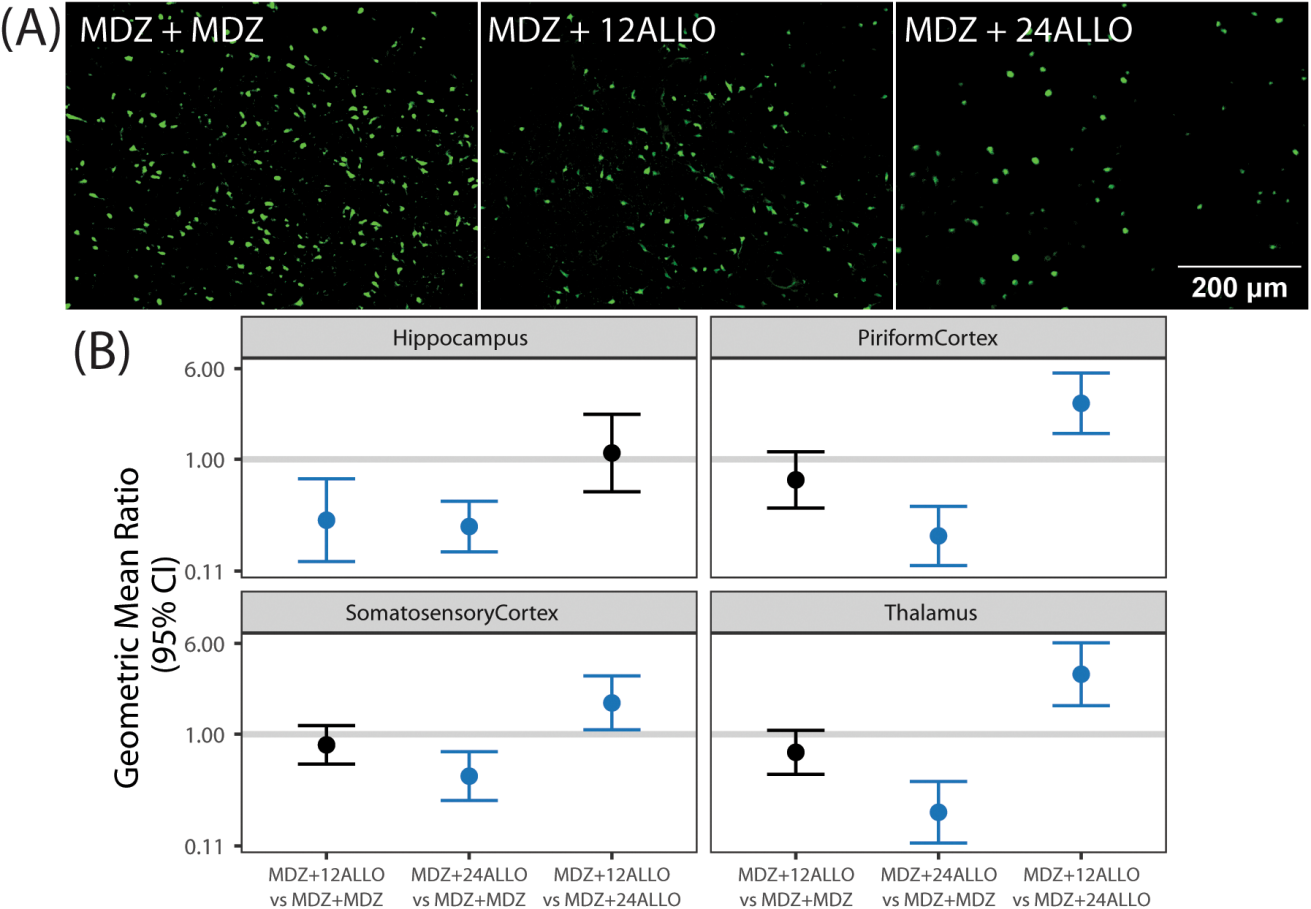
The density of degenerating neurons as identified by Fluoro‐Jade C (FJC) labelling was quantified in the somatosensory cortex, hippocampus, thalamus, piriform cortex/amygdala 4 h after exposure to soman and treatment with MDZ followed by MDZ or ALLO at 12 or 24 mg/kg. (A) Representative photomicrographs of FJC staining in the piriform cortex. (B) Geometric mean ratio (filled circles) with 95% confidence interval (CI, bar) of FJC labeled cells/mm^2^ (*n* = 6 animals per group). Confidence intervals that do not include 1.0 (indicated by the horizontal bar) indicate a statistically significant difference between the two groups being compared; bars colored blue indicate significant differences that survived false discovery rate (FDR) analysis.

Acute soman intoxication caused significant neurodegeneration at 4 h post‐exposure, as evidenced by Fluoro‐Jade C staining (Figure 4). The density of FJC‐labeled cells differed between groups and across brain region (Figure 4A,B). FJC labelling in the hippocampus was significantly decreased in both ALLO groups compared to the MDZ + MDZ group. MDZ + 24 ALLO attenuated neurodegeneration compared to MDZ + MDZ in the piriform cortex, somatosensory cortex, and thalamus (Figure 4B). The neuroprotective effect of ALLO was dose‐dependent: MDZ + 12 ALLO had significantly greater FJC labelling than MDZ + 24 ALLO in the piriform cortex, somatosensory cortex, and thalamus (Figure 4B).

**FIGURE 5:**
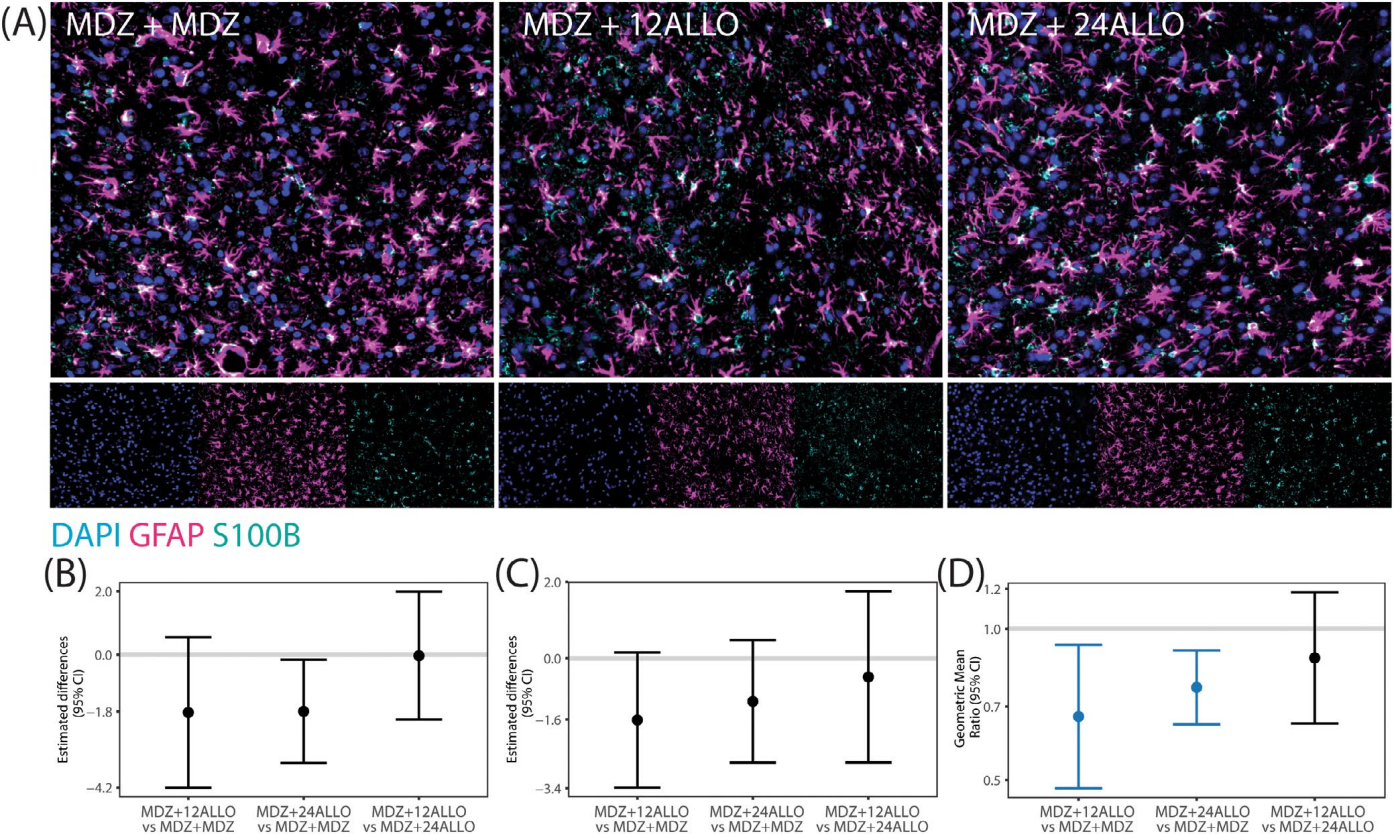
Reactive astrogliosis was evaluated by quantifying immunoreactivity of GFAP and S100β in the somatosensory cortex, hippocampus, thalamus and piriform cortex/amygdala at 4 h after exposure to soman and treatment with MDZ alone or MDZ + ALLO at 12 or 24 mg/kg. (A) Representative photomicrographs of the piriform cortex immunostained for GFAP (magenta) and S100β (cyan) to identify astrocytes; sections were counterstained with DAPI to identify cell nuclei. (B–D) Mean difference (dot) or Geometric mean ratio (GMR, dot) with 95% confidence interval (CI, bar) of (B) percent area of regions expressing GFAP, (C) percent area of regions expressing S100β, and (D) percent area expressing both GFAP and S100β (*n* = 6 animals per group). The difference between groups did not vary by brain region, so overall estimates of group differences are presented. The horizontal line in each box corresponds to a mean difference of 0 or a GMR of 1.0, which would indicate no difference between the groups. Confidence intervals that do not include 1.0 indicate a statistically significant difference between the two groups being compared; CI bars colored blue indicate the significant difference survived false discovery rate (FDR) correction.

Astrogliosis was assessed via immunolabeling with glial fibrillary acidic protein (GFAP), which is expressed largely by astrocytes within the central nervous system [37], and S100β, a protein excreted primarily by astrocytes in neuropathological conditions [38] (Figure 5A). Since the percentage of area immunopositive for GFAP and S100β did not vary significantly across brain region within any given group, all brain regions were collapsed to provide an overall estimate of the differences between groups. While the GMR values of the percentage of area immunopositive for GFAP and S100β indicated that treatment with ALLO decreased astrogliosis across all brain regions (GMRs were below 1), significant group differences were not identified for GFAP or S100β when evaluated independently (Figure 5B,C). However, treatment did significantly affect the co‐localization of GFAP/S100β (Figure 5D) with MDZ + 12 ALLO and MDZ + 24 ALLO groups having lower colocalization than the MDZ + MDZ group. There were no significant differences between the two ALLO groups.

**FIGURE 6:**
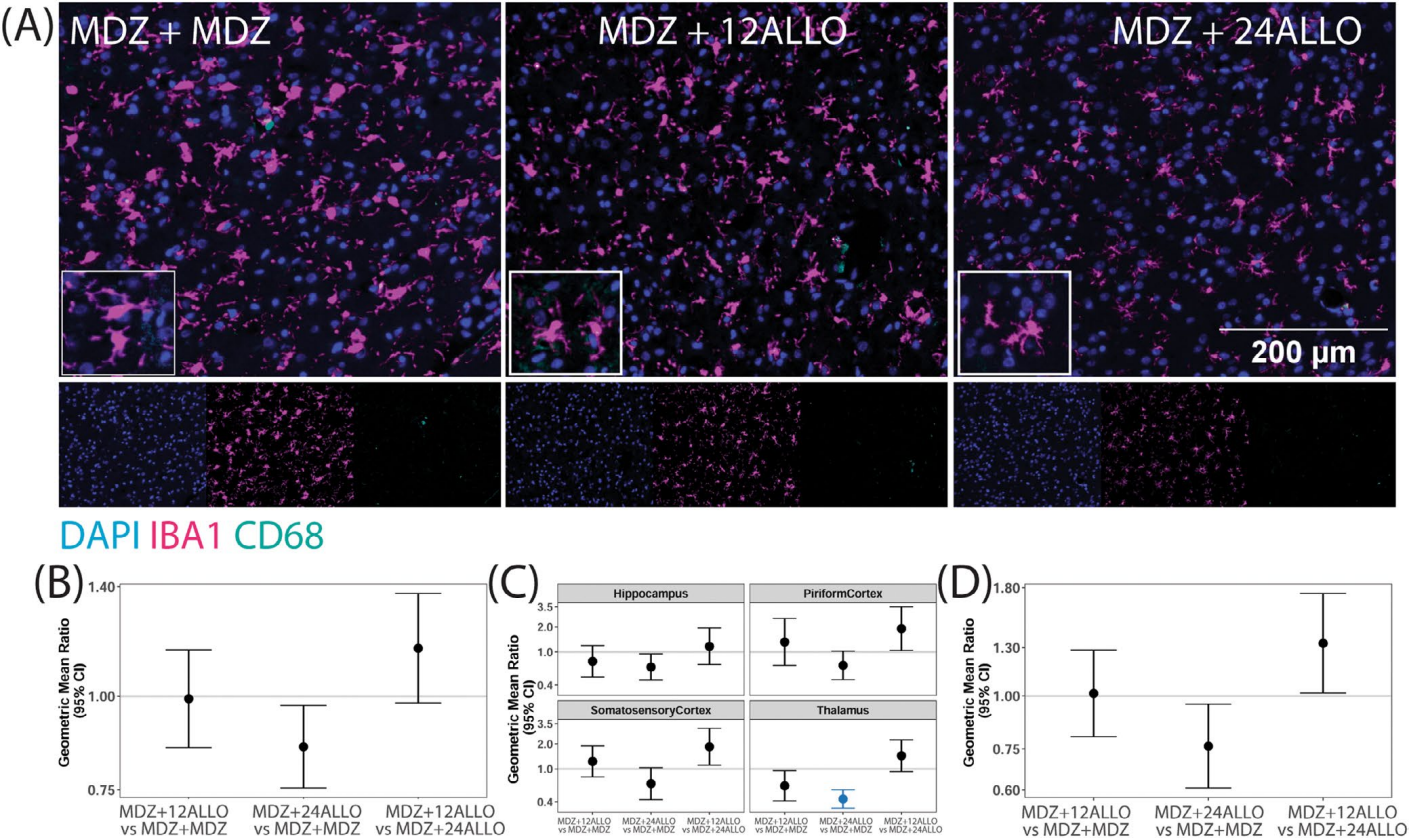
Microgliosis was evaluated by quantifying immunoreactivity of IBA1 and CD68 in the somatosensory cortex, hippocampus, thalamus and piriform cortex/amygdala 4 h after exposure to soman and treatment with MDZ alone of MDZ + ALLO at 12 or 24 mg/kg. (A) Representative photomicrographs of the piriform cortex immunostained for IBA1 (magenta) to identify microglia and CD68 (cyan) to identify phagocytic cells; sections were counterstained with DAPI to identify cell nuclei. (B–D) Geometric mean ratio (GMR, filled circles) with 95% confidence interval (CI) of (B) the percentage of total cells expressing IBA1, (C) the percentage of total cells expressing CD68, and (D) the percentage of IBA1 expressing cells also expressing CD68 (*n* = 6 animals per group). (B and D) The difference between groups did not vary by brain region, so overall estimates of group differences are presented. The horizontal line in each box corresponds to a GMR of 1.0, which would indicate the geometric mean of the parameter was identical in the two groups being compared. Confidence intervals that do not include 1.0 indicate a statistically significant difference between the two groups being compared; CI bars colored blue indicate the significant difference survived false discovery rate (FDR) correction.

Microglial responses to soman intoxication were evaluated via immunolabeling of ionized calcium binding adaptor molecule 1 (IBA1) to identify microglia [39], and cluster of differentiation 68 (CD68), a marker of phagocytic cells [40]. Consistent with previously published observations [5, 16], acute soman intoxication caused a robust microgliosis by 4 h post‐exposure (Figure 6). Differences in the percentage of IBA1 positive cells per unit area, a metric of microgliosis, did not vary significantly by brain region within groups, so an overall estimate of the treatment differences is presented. Pairwise comparisons suggested a reduction in the percentage of cells that were IBA‐1 positive in the MDZ + 24 ALLO group relative to the MDZ + MDZ group but this did not remain significant after FDR (Figure 6B). The percentage of cells expressing CD68 varied by brain region (*p* < 0.001), and the MDZ + 24 ALLO group had significantly reduced percentage of CD68^+^ cells compared to the MDZ + MDZ group in the thalamus (Figure 6C). Additional differences were found between experimental groups, but these differences did not remain significant after FDR. The extent of IBA1/CD68 colocalization also did not vary significantly by brain region, so an overall estimate of the treatment differences is presented (Figure 6D). While the GMR and CI of the comparison between MDZ + 24 ALLO versus MDZ + MDZ was less than 1, this difference did not remain significant after FDR.

## 4 Discussion

In clinical practice, SE is initially treated with a benzodiazepine such as MDZ [41]. This approach has been adapted to the treatment of OP nerve agent‐induced SE and is codified in recommendations of the U.S. Department of Health and Human Services (DHHS) on its Chemical Hazards Emergency Medical Management (CHEMM) website (https://chemm.nlm.nih.gov/). Specifically, SE suspected to be triggered by OP nerve agents is treated prehospital with a single autoinjector of MDZ (10 mg, i.m.) followed 10 min later by a second autoinjector if seizures do not cease. Our present results confirm previous reports [42] that this treatment paradigm has limited anti‐seizure efficacy in established SE associated with acute OP intoxication. In contrast, administration of ALLO as a replacement for the second dose of MDZ effectively suppressed soman‐induced SE. Critically, ALLO exhibited anti‐seizure efficacy even when administered at a delayed time after seizure initiation when MDZ was inactive. Histological analyses indicated that more effective seizure control with ALLO translates into significantly reduced neurodegeneration and perhaps attenuated neuroinflammation.

### 4.1 Combined Treatment With MDZ and ALLO Terminated Benzodiazepine‐Refractory SE

As in previous studies [42, 43], we observed that sustained SE caused by soman intoxication is benzodiazepine refractory. Specifically, in the present study, sequential treatment with two doses of MDZ at 20 and 30 min after the onset of seizures failed to terminate SE in any animals. In contrast, sequential dosing at the same treatment times with MDZ and ALLO rapidly attenuated behavioral and electrographic SE. Benzodiazepine refractoriness occurs under conditions of sustained SE [44] and is dependent on several mechanisms, including internalization of synaptic GABA_A_ receptors [44, 45], which hinders the ability of GABA_A_ receptor positive modulators such as benzodiazepines like MDZ to terminate seizures [44], and deficits in chloride extrusion capability, leading to activity‐dependent chloride accumulation that reduces the inhibitory activity of GABA_A_ receptors [19]. Neuroactive steroids like ALLO are positive allosteric modulators of not only synaptic, but also extrasynaptic GABA_A_ receptors [27]. It has been proposed that their ability to act on extrasynaptic GABA_A_ receptors differentiates them from benzodiazepines and contributes to their efficacy in the treatment of benzodiazepine‐refractory SE [20].

While our study did not evaluate the anti‐seizure efficacy of ALLO independent of MDZ, a handful of previous reports have demonstrated the efficacy of neuroactive steroids that potentiate both synaptic and extrasynaptic GABA_A_ receptors in the control of benzodiazepine‐refractory OP‐induced SE in the absence of benzodiazepine co‐therapy [32, 46]. These studies suggest that ALLO monotherapy could be effective in the treatment of OP‐induced SE without MDZ, but this requires experimental confirmation.

### 4.2 Substantially Greater Efficacy of High‐Dose ALLO

Although both the 12 and 24 mg/kg doses of ALLO suppressed soman‐induced SE, the higher dose was substantially more effective. The higher dose caused a greater suppression of behavioral SE, electrographic spiking and EEG power, and more reliably terminated SE and prevented SE recurrence. With the higher dose, SE was completely eliminated in all but one animal during the entire 4 h observation period. In contrast, most animals receiving the lower dose experienced considerable time in SE although the total time in SE was reduced for many animals. Clearly, higher brain exposures of ALLO resulting from the higher dose is likely to account for the more reliable SE suppression in the higher ALLO dose group. The lower dose of ALLO exhibited “wearing off” during the 4 h observation period that did not occur with the high dose. Plasma and brain ALLO levels fall rapidly after a bolus dose [47]. Presumably, with the low dose, brain levels fall below the threshold for seizure suppression, whereas with the high dose, brain levels may fall just as rapidly but do not fall below the threshold. If this is the case, it may be possible to maintain suppression of SE with a lower dose of ALLO by redosing at intervals.

Although ALLO suppressed electrographic seizure activity, it did not fully normalize the EEG. The lower ALLO dose reduced the EEG power and frequency of spiking, but abnormal EEG activity, including high frequency rhythmic discharges, persisted. For at least 90 min following treatment, both doses of ALLO markedly suppressed the absolute EEG power. However, aberrant ictal‐like waveforms were observed when EEG traces were inspected on a fast time base.

### 4.3 ALLO Attenuated the Acute Neurodegeneration Associated With Soman‐Induced SE

Animals receiving the higher dose of ALLO exhibited fewer degenerating neurons in all brain regions studied than the group receiving MDZ alone. Animals receiving the lower dose also had fewer degenerating neurons in all brain regions, but the reduction reached statistical significance only in the hippocampus. The high dose was significantly more effective in the somatosensory cortex, thalamus, and piriform cortex. The greater impact of the high dose may, at least in part, be due to its efficacy in preventing SE recurrence since SE recurrence has been associated with increased neurodegeneration [12, 13, 14].

Our data suggest that ALLO modulated some characteristics of acute neuroinflammatory responses triggered by acute soman intoxication. We did not observe shifts in the overall expression of astrocytic markers, GFAP or S100β, but ALLO did mitigate the expression of S100β, a mechanistic biomarker of CNS insult and an indicator of astrocytic reactivity [48], in GFAP+ cells to a greater extent than MDZ alone. ALLO did not change the percentage of IBA1‐positive cells or IBA1/CD68 colocalization. However, with high‐dose ALLO, we observed region‐specific suppression in the percentage of CD68‐positive cells, indicative of decreased phagocytic activity. These findings are consistent with published reports of a positive correlation between OP‐induced seizure duration and neuroinflammation [49]. Given that OP‐associated neuroinflammation intensifies over the days and weeks following acute OP intoxication [50], it is likely that ALLO‐mediated attenuation of soman‐induced neuroinflammation would be more evident at delayed time points.

### 4.4 Respiratory Depression Associated With High Dose ALLO

High‐dose, but not low‐dose, ALLO was associated with a transitory and self‐limited fall in average SpO_2_ during the 1 h period following treatment. The basis for the hypoxia was not investigated but, like other central nervous system depressants, is presumably due to suppression of respiratory drive by a central mechanism [51]. By themselves, benzodiazepines at recommended doses rarely cause respiratory depression, although parenteral benzodiazepines including MDZ have been shown to reduce the hypoxic ventilatory response [52]. Similarly, ALLO treatment has not been associated with respiratory depression. Indeed, in a recent study in normal dogs that intensively monitored respiratory function with physiological assessments, blood gas measurements, and recordings of SpO_2_, no adverse impact of intravenous ALLO was observed, even when administered following MDZ [53]. The maximum plasma exposures to ALLO and MDZ in this dog study were within or exceeded the ranges expected to have been obtained even with the combination including a high dose of ALLO. Thus, the animals intoxicated with soman in the present study may have been more susceptible to respiratory depression by MDZ and high‐dose ALLO than the normal dogs in our prior study. Indeed, soman is known to negatively impact respiratory function [54], which may have accounted for the increased fragility of the animals in the present study. It is also worth recognizing that the risk of respiratory depression with benzodiazepines, including MDZ, increases when used in combination with other central nervous system depressants, especially when such agents are administered at high doses [55]. In sum, the MDZ and ALLO drug combination, with ALLO at a high dose, along with the adverse respiratory effects of the toxicant soman all likely contributed to the observed hypoxia.

As far as we can tell, the hypoxia was not associated with adverse consequences. Survival was not impacted, and histological markers of brain injury were, if anything, improved by high dose ALLO in comparison with the other treatment paradigms that were not associated with hypoxia. Nevertheless, further investigation of the possible adverse consequences of ALLO‐induced transitory hypoxia is warranted. Whether administration of supplemental oxygen would be of benefit remains to be determined. As noted above, repeated administration of ALLO at a lower dose might be a strategy to obtain more complete suppression of SE with reduced risk of hypoxia.

### 4.5 Practicality of Dosing in Humans

ALLO is rapidly and completely absorbed by intramuscular administration [47]. Intramuscular injection was therefore an appropriate route of delivery for the present study. However, there is a practical limit to the volume that can be administered by intramuscular injection, with 3–5 mL considered the maximum for a single injection [56]. ALLO has limited aqueous solubility even when formulated with a cyclodextrin as was used here. We have found that intramuscular ALLO doses of 12 and 24 mg/kg are associated with peak ALLO levels of about 1000 and 2000 ng/mL, respectively (A. Dhir, C.‐Y. Wu and M.A. Rogawski, unpublished). Allometric scaling can be used to estimate the human doses that may be required to achieve these peak plasma levels. Scaling as recommended by the U.S. Food & Drug Administration suggests that the doses required may be in the range of 2 and 4 mg/kg, or 140 and 280 mg for a typical 70 kg human. Given that the ALLO concentration we used of 12 mg/mL is near the solubility limit, it is apparent that the volumes required exceed those that are practical for intramuscular administration. Therefore, dosing by an alternative route, such as intravenous or intraosseous, will likely be required to translate the present results for human application [57].

### 4.6 Impact on Long‐Term Consequences of OP Intoxication

In the present study we evaluated the impact of the treatment regimens on brain neuropathology 4 h after dosing. We did not study more clinically relevant long‐term outcomes [6, 13, 29]. There is some evidence that treatment of OP‐induced SE with GABA_A_ receptor‐positive allosteric modulator neuroactive steroids can provide enduring benefit. Thus, intervention with pregnanolone, the 5β isomer of ALLO, which has similar activity to ALLO as a positive GABA_A_ receptor modulator, attenuated neuronal cell loss at 1‐ and 3‐months post‐intoxication in a model of whole‐body sarin exposure [29]. Additional study of long‐term sequelae are warranted, with attention to possible differences between demographic groups. There is evidence that the response to acute OP intoxication and the efficacy of therapeutic interventions may differ in females or juveniles from that of adult males [58, 59]. Also, it is noteworthy that the levels of endogenous neuroactive steroids, including ALLO, differ in males and females; vary at different stages of life; fluctuate in relation to the level of stress; and, in females, fluctuate in relation to the menstrual cycle and the stage of pregnancy [60, 61]. The impact of variations in endogenous neuroactive steroids on the therapeutic activity of ALLO in the treatment of OP‐induced SE remains to be examined. Indeed, the exclusive use of male rats in this proof‐of‐concept study is a significant limitation of the current study. As recommended by the U.S. National Institutes of Health policy to study sex as a biological variable [62], follow‐up studies are critical to define efficacy parameters across a wide range of gonadal hormone statuses more representative of human populations.

## 5 Conclusions

The results of this investigation demonstrate that administration of ALLO following an initial dose of MDZ is more effective than a second dose of MDZ in the treatment of soman‐induced SE in rats. At the time of treatment in these experiments, the animals were refractory to MDZ. The initial dose of MDZ failed to suppress the seizures and a second dose was not able to overcome the refractoriness. In contrast, as previously demonstrated for other types of SE, ALLO was able to overcome the benzodiazepine refractory SE, producing a dramatic therapeutic effect. The lower 12 mg/kg dose of ALLO substantially inhibited seizures overall, but many animals exhibited signs of persistent SE, particularly at later times in the observation period. Near complete suppression of SE during the entire observation period required a higher 24 mg/kg dose of ALLO but there was transitory respiratory compromise in the first hour after dosing. The high dose of ALLO reduced the density of degenerating neurons in all brain regions examined. The low dose did not exhibit as consistent an effect on soman‐induced neuropathology but still provided a high degree of protection in the hippocampus, a particularly vulnerable area. ALLO treatment was also associated with reduced inflammatory markers. Because ALLO effectively suppressed benzodiazepine‐refractory soman‐induced SE and improved the adverse neuropathological consequences, further investigation of its use in the treatment of OP‐induced SE is warranted. In addition, ALLO may be useful more broadly in the treatment of SE due to other causes. Overall, this study supports the potential of neuroactive steroids that potentiate GABA_A_ receptors as an adjunct to benzodiazepine in the emergency treatment of SE.

## Author Contributions

**Peter M. Andrew:** data curation, investigation, writing – original draft, visualization. **Jeremy A. MacMahon:** data curation, investigation, writing – original draft, visualization. **Xiuzhen Liu:** data curation, investigation, writing – review and editing. **Naomi H. Saito:** formal analysis, writing – original draft, writing – review and editing, visualization. **Kyle E. Berger:** data curation, investigation. **Julia E. Morgan:** investigation, resources. **Ashish Dhir:** conceptualization, resources. **Danielle J. Harvey:** formal analysis, writing – original draft, writing – review and editing, visualization. **Hilary S. McCarren:** conceptualization, investigation, resources, writing – original draft, writing – review and editing, supervision, funding acquisition. **Michael A. Rogawski:** conceptualization, resources, writing – review and editing, funding acquisition. **Pamela J. Lein:** conceptualization, writing – review and editing, supervision, project administration, funding acquisition.

## Ethics Statement

All experiments involving animals were approved by the Institutional Animal Care and Use Committee (IACUC) at the United States Army Medical Research Institute of Chemical Defense. All procedures were conducted in accordance with the Guide for the Care and Use of Laboratory Animals and the Animal Welfare Act of 1966.

## Conflicts of Interest

The authors declare the following financial interests/personal relationships which may be considered as potential competing interests: A.D., P.J.L., and M.A.R. are named inventors of patents and patent applications assigned to the Regents of the University of California that are relevant to the work described here. M.A.R. serves as a consultant to Marinus Pharmaceuticals. All other authors declare no competing interests.

## Supporting information


Data S1.

## Data Availability

The data that support the findings of this study are available from the corresponding author upon reasonable request.

## References

[1] J. Newmark , “Therapy for Acute Nerve Agent Poisoning: An Update,” Neurology. Clinical Practice 9 (2019): 337–342, 10.1212/CPJ.0000000000000641. 31583189 PMC6745742

[2] H. Yamasue , O. Abe , K. Kasai , et al., “Human Brain Structural Change Related to Acute Single Exposure to Sarin,” Annals of Neurology 61 (2007): 37–46, 10.1002/ana.21024. 17187377

[3] K. Miyaki , Y. Nishiwaki , K. Maekawa , et al., “Effects of Sarin on the Nervous System of Subway Workers Seven Years After the Tokyo Subway Sarin Attack,” Journal of Occupational Health 47 (2005): 299–304, 10.1539/joh.47.299. 16096354

[4] D. A. Jett , C. A. Sibrizzi , R. B. Blain , et al., “A National Toxicology Program Systematic Review of the Evidence for Long‐Term Effects After Acute Exposure to Sarin Nerve Agent,” Critical Reviews in Toxicology 50 (2020): 474–490, 10.1080/10408444.2020.1787330. 32755358 PMC8011809

[5] F. M. de Araujo , L. A. Lumley , C. Robison , et al., “Spontaneous Recurrent Seizures After Status Epilepticus Induced by Soman in Sprague‐Dawley Rats,” Epilepsia 51 (2010): 1503–1510, 10.1111/j.1528-1167.2009.02478.x. 20067510

[6] M. Guignet , K. Dhakal , B. M. Flannery , et al., “Persistent Behavior Deficits, Neuroinflammation, and Oxidative Stress in a Rat Model of Acute Organophosphate Intoxication,” Neurobiology of Disease 133 (2020): 104431, 10.1016/j.nbd.2019.03.019. 30905768 PMC6754818

[7] E. M. Prager , V. Aroniadou‐Anderjaska , C. P. Almeida‐Suhett , et al., “The Recovery of Acetylcholinesterase Activity and the Progression of Neuropathological and Pathophysiological Alterations in the Rat Basolateral Amygdala After Soman‐Induced Status Epilepticus: Relation to Anxiety‐Like Behavior,” Neuropharmacology 81 (2014): 64–74, 10.1016/j.neuropharm.2014.01.035. 24486384 PMC4005290

[8] H. S. McCarren , M. R. Eisen , D. L. Nguyen , et al., “Characterization and Treatment of Spontaneous Recurrent Seizures Following Nerve Agent‐Induced Status Epilepticus in Mice,” Epilepsy Research 162 (2020): 106320, 10.1016/j.eplepsyres.2020.106320. 32182542 PMC7156324

[9] J. H. McDonough, Jr. , J. McMonagle , T. Copeland , D. Zoeffel , and T. M. Shih , “Comparative Evaluation of Benzodiazepines for Control of Soman‐Induced Seizures,” Archives of Toxicology 73 (1999): 473–478, 10.1007/s002040050637. 10650919

[10] J. L. Heemskerk , K. O. Abode‐Iyamah , A. Quinones‐Hinojosa , and E. S. Weinstein , “Prehospital Response Time of the Emergency Medical Service During Mass Casualty Incidents and the Effect of Triage: A Retrospective Study,” Disaster Medicine and Public Health Preparedness 16 (2022): 1091–1098, 10.1017/dmp.2021.40. 33843570

[11] C. E. Hill , A. O. Parikh , C. Ellis , J. S. Myers , and B. Litt , “Timing Is Everything: Where Status Epilepticus Treatment Fails,” Annals of Neurology 82 (2017): 155–165, 10.1002/ana.24986. 28681473 PMC5823514

[12] T. M. Shih , S. M. Duniho , and J. H. McDonough , “Control of Nerve Agent‐Induced Seizures Is Critical for Neuroprotection and Survival,” Toxicology and Applied Pharmacology 188 (2003): 69–80, 10.1016/s0041-008x(03)00019-x. 12691725

[13] B. A. Hobson , S. Siso , D. J. Rowland , et al., “From the Cover: MagneticResonance Imaging Reveals Progressive Brain Injury in Rats Acutely Intoxicated With Diisopropylfluorophosphate,” Toxicological Sciences 157 (2017): 342–353, 10.1093/toxsci/kfx049. 28329842 PMC5458789

[14] T. H. Figueiredo , J. P. Apland , M. F. M. Braga , and A. M. Marini , “Acute and Long‐Term Consequences of Exposure to Organophosphate Nerve Agents in Humans,” Epilepsia 59, no. Suppl 2 (2018): 92–99, 10.1111/epi.14500. PMC6172147 30159887

[15] D. A. Jett and S. M. Spriggs , “Translational Research on Chemical Nerve Agents,” Neurobiology of Disease 133 (2020): 104335, 10.1016/j.nbd.2018.11.020. 30468862

[16] F. M. de Araujo , F. Rossetti , S. Chanda , and D. Yourick , “Exposure to Nerve Agents: From Status Epilepticus to Neuroinflammation, Brain Damage, Neurogenesis and Epilepsy,” Neurotoxicology 33 (2012): 1476–1490, 10.1016/j.neuro.2012.09.001. 23000013

[17] H. P. Goodkin , S. Joshi , Z. Mtchedlishvili , J. Brar , and J. Kapur , “Subunit‐Specific Trafficking of GABA(A) Receptors During Status Epilepticus,” Journal of Neuroscience 28 (2008): 2527–2538, 10.1523/JNEUROSCI.3426-07.2008. 18322097 PMC2880323

[18] C. G. Wasterlain , H. Liu , D. E. Naylor , et al., “Molecular Basis of Self‐Sustaining Seizures and Pharmacoresistance During Status Epilepticus: The Receptor Trafficking Hypothesis Revisited,” Epilepsia 50, no. Suppl 12 (2009): 16–18, 10.1111/j.1528-1167.2009.02375.x. 19941513

[19] R. J. Burman , R. E. Rosch , J. M. Wilmshurst , et al., “Why Won't It Stop? The Dynamics of Benzodiazepine Resistance in Status Epilepticus,” Nature Reviews. Neurology 18 (2022): 428–441, 10.1038/s41582-022-00664-3. 35538233

[20] M. A. Rogawski , C. M. Loya , K. Reddy , D. Zolkowska , and C. Lossin , “Neuroactive Steroids for the Treatment of Status Epilepticus,” Epilepsia 54, no. Suppl 6 (2013): 93–98, 10.1111/epi.12289. 24001085 PMC3772544

[21] A. O. Rossetti , “Place of Neurosteroids in the Treatment of Status Epilepticus,” Epilepsia 59, no. Suppl 2 (2018): 216–219, 10.1111/epi.14481. 30159866

[22] I. Younus and D. S. Reddy , “A Resurging Boom in New Drugs for Epilepsy and Brain Disorders,” Expert Review of Clinical Pharmacology 11 (2018): 27–45, 10.1080/17512433.2018.1386553. 28956955

[23] N. Walton and J. Maguire , “Allopregnanolone‐Based Treatments for Postpartum Depression: Why/How Do They Work?,” Neurobiology of Stress 11 (2019): 100198, 10.1016/j.ynstr.2019.100198. 31709278 PMC6838978

[24] M. S. Saporito , J. A. Gruner , A. DiCamillo , R. Hinchliffe , M. Barker‐Haliski , and H. S. White , “Intravenously Administered Ganaxolone Blocks Diazepam‐Resistant Lithium‐Pilocarpine‐Induced Status Epilepticus in Rats: Comparison With Allopregnanolone,” Journal of Pharmacology and Experimental Therapeutics 368 (2019): 326–337, 10.1124/jpet.118.252155. 30552296

[25] E. Broomall , J. E. Natale , M. Grimason , et al., “Pediatric Super‐Refractory Status Epilepticus Treated With Allopregnanolone,” Annals of Neurology 76 (2014): 911–915, 10.1002/ana.24295. 25363147 PMC4534165

[26] A. Dhir , D. A. Bruun , M. Guignet , et al., “Allopregnanolone and Perampanel as Adjuncts to Midazolam for Treating Diisopropylfluorophosphate‐Induced Status Epilepticus in Rats,” Annals of the New York Academy of Sciences 1480 (2020): 183–206, 10.1111/nyas.14479. 32915470 PMC7756871

[27] E. Gilat , T. Kadar , A. Levy , et al., “Anticonvulsant Treatment of Sarin‐Induced Seizures With Nasal Midazolam: An Electrographic, Behavioral, and Histological Study in Freely Moving Rats,” Toxicology and Applied Pharmacology 209 (2005): 74–85, 10.1016/j.taap.2005.03.007. 16271623

[28] B. S. Barker , J. Spampanato , H. S. McCarren , et al., “Screening for Efficacious Anticonvulsants and Neuroprotectants in Delayed Treatment Models of Organophosphate‐Induced Status Epilepticus,” Neuroscience 425 (2020): 280–300, 10.1016/j.neuroscience.2019.11.020. 31783100 PMC6935402

[29] L. Lumley , D. Miller , W. T. Muse , et al., “Neurosteroid and Benzodiazepine Combination Therapy Reduces Status Epilepticus and Long‐Term Effects of Whole‐Body Sarin Exposure in Rats,” Epilepsia Open 4 (2019): 382–396, 10.1002/epi4.12344. 31440720 PMC6698686

[30] D. A. Nguyen , M. F. Stone , C. R. Schultz , et al., “Evaluation of Midazolam‐Ketamine‐Allopregnanolone Combination Therapy Against Cholinergic‐Induced Status Epilepticus in Rats,” Journal of Pharmacology and Experimental Therapeutics 388 (2024): 376–385, 10.1124/jpet.123.001784. 37770198 PMC10801769

[31] A. Rojas , H. S. McCarren , J. Wang , et al., “Comparison of Neuropathology in Rats Following Status Epilepticus Induced by Diisopropylfluorophosphate and Soman,” Neurotoxicology 83 (2021): 14–27, 10.1016/j.neuro.2020.12.010. 33352274 PMC7987879

[32] A. L. Althaus , H. S. McCarren , A. Alqazzaz , et al., “The Synthetic Neuroactive Steroid SGE‐516 Reduces Status Epilepticus and Neuronal Cell Death in a Rat Model of Soman Intoxication,” Epilepsy & Behavior 68 (2017): 22–30, 10.1016/j.yebeh.2016.12.024. 28109985

[33] L. C. Schmued , C. C. Stowers , A. C. Scallet , and L. Xu , “Fluoro‐Jade C Results in Ultra High Resolution and Contrast Labeling of Degenerating Neurons,” Brain Research 1035 (2005): 24–31, 10.1016/j.brainres.2004.11.054. 15713273

[34] L. J. Hirsch , M. W. K. Fong , M. Leitinger , et al., “American Clinical Neurophysiology Society's Standardized Critical Care EEG Terminology: 2021 Version,” Journal of Clinical Neurophysiology 38 (2021): 1–29, 10.1097/WNP.0000000000000806. 33475321 PMC8135051

[35] L. S. Deshpande , D. S. Carter , R. E. Blair , and R. J. DeLorenzo , “Development of a Prolonged Calcium Plateau in Hippocampal Neurons in Rats Surviving Status Epilepticus Induced by the Organophosphate Diisopropylfluorophosphate,” Toxicological Sciences 116 (2010): 623–631, 10.1093/toxsci/kfq157. 20498005 PMC2905411

[36] P. L. Nunez and R. Srinivasan , Electric Fields of the Brain: The Neurophysics of EEG (New York, NY: Oxford University Press, 2006).

[37] Z. Yang and K. K. Wang , “Glial Fibrillary Acidic Protein: From Intermediate Filament Assembly and Gliosis to Neurobiomarker,” Trends in Neurosciences 38 (2015): 364–374, 10.1016/j.tins.2015.04.003. 25975510 PMC4559283

[38] F. Michetti , G. Di Sante , M. E. Clementi , et al., “Growing Role of S100B Protein as a Putative Therapeutic Target for Neurological‐ and Nonneurological‐Disorders,” Neuroscience and Biobehavioral Reviews 127 (2021): 446–458, 10.1016/j.neubiorev.2021.04.035. 33971224

[39] Y. Sasaki , K. Ohsawa , H. Kanazawa , S. Kohsaka , and Y. Imai , “Iba1 Is an Actin‐Cross‐Linking Protein in Macrophages/Microglia,” Biochemical and Biophysical Research Communications 286 (2001): 292–297, 10.1006/bbrc.2001.5388. 11500035

[40] D. A. Chistiakov , M. C. Killingsworth , V. A. Myasoedova , A. N. Orekhov , and Y. V. Bobryshev , “CD68/Macrosialin: Not Just a Histochemical Marker,” Laboratory Investigation 97 (2017): 4–13, 10.1038/labinvest.2016.116. 27869795

[41] G. M. Brophy , R. Bell , J. Claassen , et al., “Guidelines for the Evaluation and Management of Status Epilepticus,” Neurocritical Care 17 (2012): 3–23, 10.1007/s12028-012-9695-z. 22528274

[42] X. Wu , R. Kuruba , and D. S. Reddy , “Midazolam‐Resistant Seizures and Brain Injury After Acute Intoxication of Diisopropylfluorophosphate, an Organophosphate Pesticide and Surrogate for Nerve Agents,” Journal of Pharmacology and Experimental Therapeutics 367 (2018): 302–321, 10.1124/jpet.117.247106. 30115757 PMC6193253

[43] H. S. McCarren and J. H. McDonough, Jr. , “Anticonvulsant Discovery Through Animal Models of Status Epilepticus Induced by Organophosphorus Nerve Agents and Pesticides,” Annals of the New York Academy of Sciences 1374 (2016): 144–150, 10.1111/nyas.13092. 27258770

[44] J. Niquet , R. Baldwin , L. Suchomelova , et al., “Benzodiazepine‐Refractory Status Epilepticus: Pathophysiology and Principles of Treatment,” Annals of the New York Academy of Sciences 1378 (2016): 166–173, 10.1111/nyas.13147. 27392038 PMC5063678

[45] T. Z. Deeb , J. Maguire , and S. J. Moss , “Possible Alterations in GABAA Receptor Signaling That Underlie Benzodiazepine‐Resistant Seizures,” Epilepsia 53, no. Suppl 9 (2012): 79–88, 10.1111/epi.12037. PMC4402207 23216581

[46] T. B. C. Johnstone , H. S. McCarren , J. Spampanato , et al., “Enaminone Modulators of Extrasynaptic α_4_β_3_δ γ‐minobutyric AcidA Receptors Reverse Electrographic Status Epilepticus in the Rat After Acute Organophosphorus Poisoning,” Frontiers in Pharmacology 10 (2019): 560, 10.3389/fphar.2019.00560. 31178732 PMC6543275

[47] D. Zolkowska , C. Y. Wu , and M. A. Rogawski , “Intramuscular Allopregnanolone and Ganaxolone in a Mouse Model of Treatment‐Resistant Status Epilepticus,” Epilepsia 59, no. Suppl 2 (2018): 220–227, 10.1111/epi.13999. 29453777 PMC6910080

[48] F. Michetti , N. D'Ambrosi , A. Toesca , et al., “The S100B Story: From Biomarker to Active Factor in Neural Injury,” Journal of Neurochemistry 148 (2019): 168–187, 10.1111/jnc.14574. 30144068

[49] S. Sisó , B. A. Hobson , D. J. Harvey , et al., “Editor's Highlight: Spatiotemporal Progression and Remission of Lesions in the Rat Brain Following Acute Intoxication With Diisopropylfluorophosphate,” Toxicological Sciences 157 (2017): 330–341, 10.1093/toxsci/kfx048. 28329845 PMC6070115

[50] P. M. Andrew and P. J. Lein , “Neuroinflammation as a Therapeutic Target for Mitigating the Long‐Term Consequences of Acute Organophosphate Intoxication,” Frontiers in Pharmacology 12 (2021): 674325, 10.3389/fphar.2021.674325. 34054549 PMC8153682

[51] A. Forster , J. P. Gardaz , P. M. Suter , and M. Gemperle , “Respiratory Depression by Midazolam and Diazepam,” Anesthesiology 53 (1980): 494–497, 10.1097/00000542-198012000-00010. 7457966

[52] K. H. Mak , Y. T. Wang , T. H. Cheong , and S. C. Poh , “The Effect of Oral Midazolam and Diazepam on Respiration in Normal Subjects,” European Respiratory Journal 6 (1993): 42–47. 8425593

[53] D. A. Bruun , B. Ma , Y. J. Chen , et al., “Tolerability and Pharmacokinetics of Intravenous Allopregnanolone With and Without Midazolam Pretreatment in Two Healthy Dogs,” Epilepsia Open 8 (2023): 666–672, 10.1002/epi4.12723. 36919379 PMC10235566

[54] E. J. Hulse , J. O. Davies , A. J. Simpson , A. M. Sciuto , and M. Eddleston , “Respiratory Complications of Organophosphorus Nerve Agent and Insecticide Poisoning. Implications for Respiratory and Critical Care,” American Journal of Respiratory and Critical Care Medicine 190 (2014): 1342–1354, 10.1164/rccm.201406-1150CI. 25419614 PMC4299648

[55] T. N. Lingamchetty , S. A. Hosseini , and A. Saadabadi , “Midazolam. In "StatPearls" Treasure Island (FL) With Ineligible Companies. Disclosure: Seyed Alireza Hosseini Declares No Relevant Financial Relationships With Ineligible Companies. Disclosure: Abdolreza Saadabadi Declares No Relevant Financial Relationships With Ineligible Companies,” 2024.

[56] M. A. Rodger and L. King , “Drawing Up and Administering Intramuscular Injections: A Review of the Literature,” Journal of Advanced Nursing 31 (2000): 574–582, 10.1046/j.1365-2648.2000.01312.x. 10718876

[57] J. P. Orlowski , D. T. Porembka , J. M. Gallagher , J. D. Lockrem , and F. VanLente , “Comparison Study of Intraosseous, Central Intravenous, and Peripheral Intravenous Infusions of Emergency Drugs,” American Journal of Diseases of Children 144 (1990): 112–117, 10.1001/archpedi.1990.02150250124049. 1688484

[58] E. A. Gonzalez , J. J. Calsbeek , Y. H. Tsai , et al., “Sex‐Specific Acute and Chronic Neurotoxicity of Acute Diisopropylfluorophosphate (DFP)‐Intoxication in Juvenile Sprague‐Dawley Rats,” Current Research in Toxicology 2 (2021): 341–356, 10.1016/j.crtox.2021.09.002. 34622217 PMC8484742

[59] M. Gage , M. Golden , M. Putra , S. Sharma , and T. Thippeswamy , “Sex as a Biological Variable in the Rat Model of Diisopropylfluorophosphate‐Induced Long‐Term Neurotoxicity,” Annals of the New York Academy of Sciences 1479 (2020): 44–64, 10.1111/nyas.14315. 32090337 PMC7483197

[60] S. Kanes , H. Colquhoun , H. Gunduz‐Bruce , et al., “Brexanolone (SAGE‐547 Injection) in Post‐Partum Depression: A Randomised Controlled Trial,” Lancet 390 (2017): 480–489, 10.1016/S0140-6736(17)31264-3. 28619476

[61] F. Bidlingmaier , M. Wagner‐Barnack , O. Butenandt , and D. Knorr , “Plasma Estrogens in Childhood and Puberty Under Physiologic and Pathologic Conditions,” Pediatric Research 7 (1973): 901–907, 10.1203/00006450-197311000-00006. 4795966

[62] C. A. Christian‐Hinman , “The Promise and Practicality of Addressing Sex as a Biological Variable and the Ovarian Cycle in Preclinical Epilepsy Research,” Epilepsy Currents 24 (2024): 274–279, 10.1177/15357597241261463. 39309055 PMC11412390

