# Supplementary material for: Allopregnanolone as an Adjunct Therapy to Midazolam is More Effective Than Midazolam Alone in Suppressing Soman‐Induced Status Epilepticus in Male Rats
Source: CNS Neurosci Ther. 2025 Feb 28;31(3):e70215. doi: 10.1111/cns.70215 (PMC11871396; doi:10.1111/cns.70215)
Supplement: Supplementary file 1 — Data S1. [file CNS-31-e70215-s001.docx]

**Supplemental Table 1.** Primary and secondary antibodies used for quantitative immunohistochemistry

| **Antibody** | **Dilution** | **Source/Catalog #** | **RRID** |
| --- | --- | --- | --- |
| Mouse anti-GFAP | 1:1000 | Cell Signaling Technology/ #3670 | AB_561076 |
| Rabbit anti-S100β | 1:300 | Abcam/# ab52642 | AB_882426 |
| Rabbit anti-IBA1 | 1:1000 | Wako Laboratory Chemicals/ 019–19741 | AB_839504 |
| Rat anti-CD68 | 1:200 | Bio-Rad/ MCA1957 | AB_322219 |
| Goat anti-rabbit IgG Alexa Fluor 568 | 1:1000 | Life Technologies/ A11036 | AB_1056566 |
| Goat anti-mouse IgG1 Alexa Fluor 568 | 1:1000 | Thermo Fisher Scientific/ A21124 | AB_2535766 |
| Goat anti-rat IgG Alexa Fluor 488 | 1:500 | Thermo Fisher Scientific/ A11006 | AB_2534074 |
| Goat anti-rabbit Alexa Fluor 488 | 1:600 | Thermo Fisher Scientific/ A11034 | AB_2576217 |
|  |  |  |  |
|  |  |  |  |

**Supplemental Table 2.** Geometric Mean Ratios for Group comparisons of EEG spiking. Bolded values remained significant after FDR correction

|  | **MDZ + 12 ALLO**  **vs**  **MDZ + MDZ** | | | **MDZ + 24 ALLO**  **vs**  **MDZ + MDZ** | | | **MDZ + 12 ALLO**  **vs**  **MDZ + 24 ALLO** | | |
| --- | --- | --- | --- | --- | --- | --- | --- | --- | --- |
|  | GMR | 95% CI | p-value | GMR | 95% CI | p-value | GMR | 95% CI | p-value |
| Baseline | 1.4 | 0.6-2.9 | .42 | 1.1 | 0.4-2.9 | .84 | 1.2 | 0.5-2.8 | .62 |
| Post Soman | 0.9 | 0.8-1.1 | .30 | 1.0 | 0.8-1.3 | .77 | 0.9 | 0.7-1.0 | .13 |
| Post MDZ | 1.0 | 0.8-1.1 | .58 | 0.9 | 0.7-1.1 | .26 | 1.1 | 0.9-1.2 | .40 |
| Post 2^nd^ treatment | **0.25** | **0.18-0.36** | **<.001** | **0.06** | **0.04-0.10** | **<.001** | **4.0** | **2.5-6.6** | **<.001** |
| Late treatment | **0.07** | **0.01-0.36** | **.002** | **0.006** | **0.003-0.011** | **<.001** | **11.9** | **2.2-64.0** | **.004** |

**Supplemental Table 3.** Geometric Mean Ratios for Group comparisons of EEG power. Bolded values remained significant after FDR correction

|  | **MDZ + 12 ALLO**  **vs**  **MDZ + MDZ** | | | **MDZ + 24 ALLO**  **vs**  **MDZ + MDZ** | | | **MDZ + 12 ALLO**  **vs**  **MDZ + 24 ALLO** | | |
| --- | --- | --- | --- | --- | --- | --- | --- | --- | --- |
|  | GMR | 95% CI | p-value | GMR | 95% CI | p-value | GMR | 95% CI | p-value |
| Baseline | 1.1 | 08-1.4 | .76 | 0.9 | 0.7-1.3 | .73 | 1.1 | 0.8-1.5 | .46 |
| Post Soman | 1.5 | 0.9-2.6 | .10 | 0.8 | 0.6-1.3 | .43 | **1.8** | **1.2-2.8** | **.004** |
| Post MDZ | 1.3 | 0.8-2.1 | .23 | 0.7 | 0.4-1.0 | .03 | **2.0** | **1.3-3.2** | **.003** |
| Post 2^nd^ treatment | **0.4** | **0.2-0.6** | **<.001** | **0.06** | **0.03-0.09** | **<.001** | **6.7** | **4.0-11.2** | **<.001** |
| Late treatment | **0.3** | **0.2-0.7** | **.004** | **0.08** | **0.06-0.11** | **<.001** | **4.0** | **2.0-8.0** | **<.001** |

**Supplemental Table 4.** Geometric Mean Ratios for Group comparisons of density of FJC labeled cells. Bolded values remained significant after FDR correction

|  | **MDZ + 12 ALLO**  **vs**  **MDZ + MDZ** | | | **MDZ + 24 ALLO**  **vs**  **MDZ + MDZ** | | | **MDZ + 12 ALLO**  **vs**  **MDZ + 24 ALLO** | | |
| --- | --- | --- | --- | --- | --- | --- | --- | --- | --- |
|  | GMR | 95% CI | p-value | GMR | 95% CI | p-value | GMR | 95% CI | p-value |
| Hippo-  campus | **0.3** | **0.1-0.7** | **.005** | **0.3** | **0.2-0.4** | **<.001** | 1.1 | 0.5-2.4 | .74 |
| Piriform  Cortex/  Amygdala | 0.7 | 0.4-1.2 | .15 | **0.2** | **0.1-0.4** | **<.001** | **3.0** | **1.7-5.5** | **<.001** |
| Somato-  sensory  Cortex | 0.8 | 0.6-1.2 | .27 | **0.4** | **0.3-0.7** | **0.001** | **1.9** | **1.1-3.2** | **.02** |
| Thalamus | 0.7 | 0.5-1.1 | .10 | **0.2** | **0.1-0.4** | **<.001** | **3.3** | **1.8-6.1** | **<.001** |

**Supplemental Table 5.** Mean Differences or Geometric Mean Ratios for Group comparisons of GFAP, S100β, GFAP/S100β colocalization. Bolded values remained significant after FDR correction

|  | **MDZ + 12 ALLO**  **vs**  **MDZ + MDZ** | | | **MDZ + 24 ALLO**  **vs**  **MDZ + MDZ** | | | **MDZ + 12 ALLO**  **vs**  **MDZ + 24 ALLO** | | |
| --- | --- | --- | --- | --- | --- | --- | --- | --- | --- |
|  | Mean difference or GMR* | 95% CI | p-value | Mean difference or GMR* | 95% CI | p-value | Mean difference or GMR* | 95% CI | p-value |
| GFAP | -1.8 | -4.2-0.5 | .12 | -1.8 | -3.4- -0.2 | .03 | -0.03 | -2.1-2.0 | .97 |
| S100β | -1.6 | -3.4-0.2 | .07 | -1.1 | -2.7-0.5 | .16 | -0.5 | -2.7-1.7 | .65 |
| GFAP/  S100β | **0.7** | **0.5-0.9** | **.02** | **0.8** | **0.6-0.9** | **.004** | 0.9 | 0.6-1.2 | .36 |

*Mean difference is presented for GFAP and S100β, while GMR is presented for GFAP/S100β.

**Supplemental Table 6.** Geometric Mean Ratios for Group comparisons of IBA1 and IBA1/CD68 colocalization

|  | **MDZ + 12 ALLO**  **vs**  **MDZ + MDZ** | | | **MDZ + 24 ALLO**  **vs**  **MDZ + MDZ** | | | **MDZ + 12 ALLO**  **vs**  **MDZ + 24 ALLO** | | |
| --- | --- | --- | --- | --- | --- | --- | --- | --- | --- |
|  | GMR | 95% CI | p-value | GMR | 95% CI | p-value | GMR | 95% CI | p-value |
| IBA1 | 1.0 | 0.9-1.2 | .92 | 0.86 | 0.75-0.97 | .02 | 1.2 | 1.0-1.4 | .08 |
| IBA1/CD68 | 1.0 | 0.8-1.3 | .90 | 0.78 | 0.61-0.96 | .02 | 1.3 | 1.0-1.7 | .04 |

**Supplemental Table 7.** Geometric Mean Ratios for Group comparisons of CD68. Bolded values remained significant after FDR correction

|  | **MDZ + 12 ALLO**  **vs**  **MDZ + MDZ** | | | **MDZ + 24 ALLO**  **vs**  **MDZ + MDZ** | | | **MDZ + 12 ALLO**  **vs**  **MDZ + 24 ALLO** | | |
| --- | --- | --- | --- | --- | --- | --- | --- | --- | --- |
|  | GMR | 95% CI | p-value | GMR | 95% CI | p-value | GMR | 95% CI | p-value |
| Hippo-  campus | 0.8 | 0.5-1.2 | .24 | 0.7 | 0.5-0.9 | .03 | 1.2 | 0.7-1.9 | .53 |
| Piriform  Cortex/  Amygdala | 1.3 | 0.7-2.5 | .39 | 0.7 | 0.5-1.0 | .07 | 1.9 | 1.0-3.5 | .04 |
| Somato-  sensory  Cortex | 1.2 | 0.8-1.9 | .34 | 0.7 | 0.4-1.0 | .07 | 1.8 | 1.1-3.1 | .02 |
| Thalamus | 0.6 | 0.4-1.0 | .03 | **0.4** | **0.3-0.6** | **<.001** | 1.4 | 0.9-2.2 | .10 |
